# Pilot Study on the Use of a Laser-Structured Double Diamond Electrode (DDE) for Biofilm Removal from Dental Implant Surfaces

**DOI:** 10.3390/jcm9093036

**Published:** 2020-09-21

**Authors:** Maximilian Koch, Andreas Burkovski, Manuel Zulla, Stefan Rosiwal, Walter Geißdörfer, Roman Dittmar, Tanja Grobecker-Karl

**Affiliations:** 1 Microbiology Division, Department of Biology, University of Erlangen-Nuremberg, 91058 Erlangen, Germany; max.koch@fau.de; 2 Division of Ultra-Hard Coatings, Department of Material Sciences, University of Erlangen-Nuremberg, 91058 Erlangen, Germany; m.zulla@outlook.com; 3 Chair of Materials Science and Engineering for Metals, Department of Material Sciences, University of Erlangen-Nuremberg, 91058 Erlangen, Germany; Stefan.Rosiwal@fau.de; 4 Institute of Clinical Microbiology, Immunology and Hygiene, Universitätsklinikum Erlangen, Friedrich-Alexander-Universität Erlangen-Nürnberg, 91054 Erlangen, Germany; Walter.Geissdoerfer@uk-erlangen.de; 5 Institut Straumann AG, 4052 Basel, Switzerland; roman.dittmar@straumann.com; 6 Department of Prosthodontics, Saarland University, 66421 Homburg/Saar, Germany; tanja.grobecker-karl@uks.eu

**Keywords:** boron-doped diamond, chemo-mechanical treatment, electrochemical disinfection, peri-implantitis

## Abstract

No proper treatment option for peri-implantitis exists yet. Based on previous studies showing the in vitro effectiveness of electrochemical disinfection using boron-doped diamond electrodes, novel double diamond electrodes (DDE) were tested here. Using a ceramic carrier and a laser structuring process, a clinically applicable electrode array was manufactured. Roughened metal discs (*n* = 24) made from Ti-Zr alloy were exposed to the oral cavities of six volunteers for 24 h in order to generate biofilm. Then, biofilm removal was carried out either using plastic curettes and chlorhexidine digluconate or electrochemical disinfection. In addition, dental implants were contaminated with ex vivo multispecies biofilm and disinfected using DDE treatment. Bacterial growth and the formation of biofilm polymer were determined as outcome measures. Chemo-mechanical treatment could not eliminate bacteria from roughened surfaces, while in most cases, a massive reduction of bacteria and biofilm polymer was observed following DDE treatment. Electrochemical disinfection was charge- and time-dependent and could also not reach complete disinfection in all instances. Implant threads had no negative effect on DDE treatment. Bacteria exhibit varying resistance to electrochemical disinfection with *Bacillus subtilis*, *Neisseria* sp., *Rothia* *mucilaginosa*, *Staphylococcus haemolyticus*, and *Streptococcus mitis* surviving 5 min of DDE application at 6 V. Electrochemical disinfection is promising but requires further optimization with respect to charge quantity and application time in order to achieve disinfection without harming host tissue.

## 1. Introduction

Peri-implantitis is currently understood as an inflammatory process affecting soft and hard tissues surrounding dental implants [1] and resulting in the destruction of alveolar bone and attachment [2]. As such, peri-implantitis has to be differentiated from adaptation processes occurring as a result of surgical trauma and loading, leading to foreign body equilibrium [3].

Potentially due to improper definition over a period of several years as well as the fact that several patient- and implant-related factors have to be considered clinically [1], the prevalence of peri-implantitis reported differs vastly among authors. Jepsen and coworkers described a peri-implant mucositis prevalence of 43% and peri-implantitis prevalence of 22% [4], while Rakic and coworkers found a peri-implantitis prevalence of 18.5% at patient level and 12.8% at implant level [5]. In contrast to that, Albrektsson and coworkers found only 1 to 2% of implants showing true peri-implantitis during follow-up periods of 10 years or more [6] and 2.7% of implants requiring surgical intervention during 7 to 16 years of function [7].

There seems to be consensus that plaque accumulation, bacterial pathogens, and immunological reactions play an important role in the pathogenesis of peri-implantitis [4,6,8,9], and consequently, several treatment modalities [10] have been described. These options range from mechanical debridement, e.g., using a titanium brush [11] followed by the application of chlorhexidine [12,13] or 35% phosphoric acid [14] to air powder treatment with sodium carbonate [15], which may also be combined with local antibiotics or antiseptics [10]. Air-polishing with 40 µm bicarbonate powder has just recently been shown to be more efficient in biofilm removal as compared to the use of a nickel–titanium brush, leaving the implant surface conducive for cell adhesion [16,17]. Advanced strategies include ozone therapy [18], photodynamic therapy [19], the use of lasers [20,21], and cold atmospheric plasma [22]. Based on comparative studies, it appears that the debridement method itself only has a minor impact [20,21] and that the evidence on the efficacy of non-surgical and surgical therapies in the treatment of peri-implantitis is limited [19,23]. Maintenance care for implant patients including the non-surgical treatment of peri-implantitis prior to surgical intervention has to be stressed [24].

The major challenges in peri-implantitis therapy include the risk of changes in implant surface topography [25] due to instrumentation as well as limited access depending on the morphology of the peri-implant defect [26]. Two recent studies pointed out that the complete removal of biofilms from implant surfaces is not feasible due to the macrodesign of the implants [25,27]. As a result of these limitations, the removal of biofilms based on the electrolysis of water has been advocated [28] as an alternative. However, when osseointegrated implants are being used as electrodes for this process of generating mechanically acting gas bubbles, the risk of hydrogen embrittlement of titanium exists [29,30]. 

A novel approach for disinfecting hardly accessible porous structures employs the use of boron-doped diamond (BDD) electrodes and has been derived from wastewater treatment [31]. Previous investigations using simplistic arrays of single electrodes have shown that electrochemical disinfection can effectively inactivate monospecies biofilm both during root canal treatment [32] and peri-implantitis treatment [33] without affecting surface characteristics, as may be the case with the use of curettes and airflow instruments [34]. Further developing this technology for clinical application, it was the goal of this study to fabricate and test a novel laser-structured, ceramic-based BDD electrode array for removing wild-type multispecies biofilm.

## 2. Experimental Section

### 2.1. Intraoral Formation of Wild-Type Biofilm

**Figure 1:**
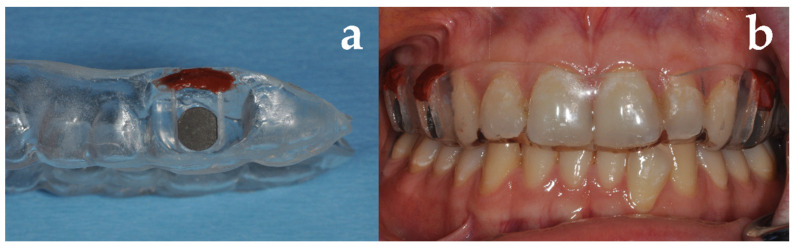
Maxillary splint with recesses for mounting Ti-Zr discs. (**a**) Recess used for mounting the Ti-Zr discs allowing for easy removal, (**b**) intraoral situation with splint in place.

Following written consent, six volunteers with natural dentition but unknown periodontal status received maxillary splints onto each of which four Ti-Zr discs (5 mm in diameter, 1 mm thickness, Institut Straumann AG, Basel, Switzerland) were mounted (Figure 1) and exposed to the volunteers’ oral cavity for 24 h. The roughened surfaces of the discs were facing the vestibule, while the smooth surfaces were oriented toward the teeth. The volunteers were allowed to remove the splints during eating, temporarily storing them on wet gauze, but they were not allowed to perform any oral hygiene measures. 

### 2.2. Electrochemical Disinfection

#### 2.2.1. Boron-Doped Double Diamond Electrode

**Figure 2:**
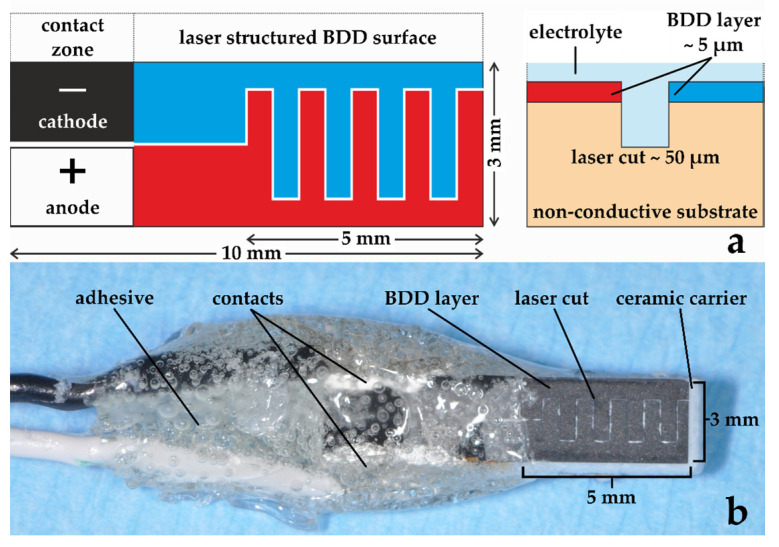
Ceramic double diamond electrode. The conductive boron-doped diamond (BDD) layer is added to a non-conductive carrier. The laser cut allows both electrodes to share the same carrier and reduces the gap between the anode and cathode. (**a**) Schematic representation, (**b**) prototype.

The double diamond electrode (DDE) used for disinfection in this study is based on the unique electrochemical behavior of boron-doped diamond (BDD) electrodes producing oxidative reagents at the anodic surface, but it differs significantly from the state of the art of BDD in literature. In general, the diamond coating is performed on metallic substrates (W, Nb, Ti) as bulk material with thin interlayers (e.g., TiC or TiN) on top for better adhesion [32,33,34,35,36]. In this newly developed double diamond system, both the anode and cathode consist of a BDD layer, which is deposited to a non-conductive ceramic substrate (porcelain, Al_4_[(OH)_8_|Si_4_O_10_], (Ca,Na,K)(Al,Si)_4_O_8_, SiO_2_, Flügel Porzellan, Selb, Germany) by Chemical Vapor Deposition (CVD). The diamond coating is performed by a standard hot filament CVD process [32,33,34,35]. Due to the addition of B(OCH_3_)_3_ gas during the coating process, the diamond layer becomes conductive (doping), while the substrate stays non-conductive. This unique configuration of DDEs enables a variety of new options because the coated component can be electrically separated with laser structuring (Figure 2), enabling a high production rate of hydroxyl radicals and other oxidative species at the anodic BDD surface despite the small dimensions of the whole electrode.

A copper cable strand (0.14 mm^2^) was attached to each side as an electrical contact. Conductive silver paint (Silberleitlack, Ferro GmbH, Frankfurt/Main, Germany) was used as an adhesion promoter for the diamond surface and the copper strand. After drying, the contact zone was insulated with glue (UHU 44510 Hart Spezialkleber, Bühl, Germany) to protect it from a potential negative influence from the reactive zone. 

#### 2.2.2. Treatment of Discs

**Figure 3:**
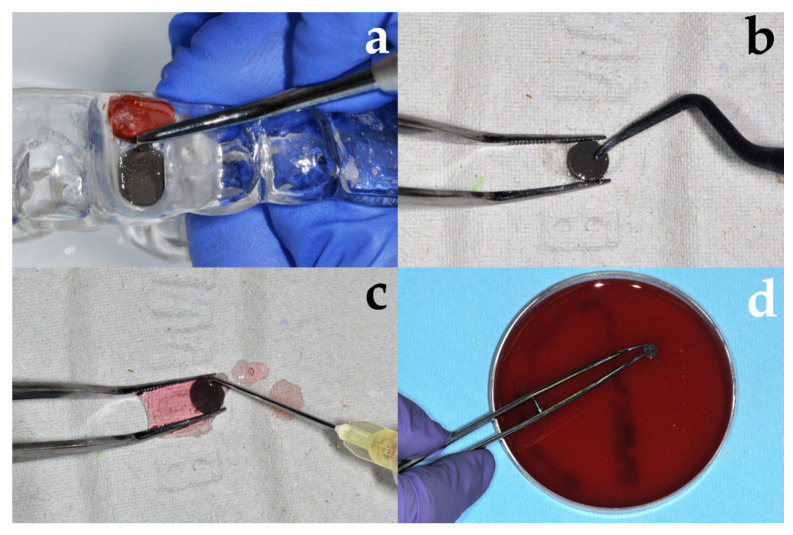
Chemo-mechanical debridement of Ti-Zr discs following biofilm formation. (**a**) Harvesting of the disc from the splint, (**b**) treatment with plastic curette, (**c**) rinsing with chlorhexidine, (**d**) samples repeatedly pressed with roughened and smooth surface on blood agar plate.

**Figure 4:**
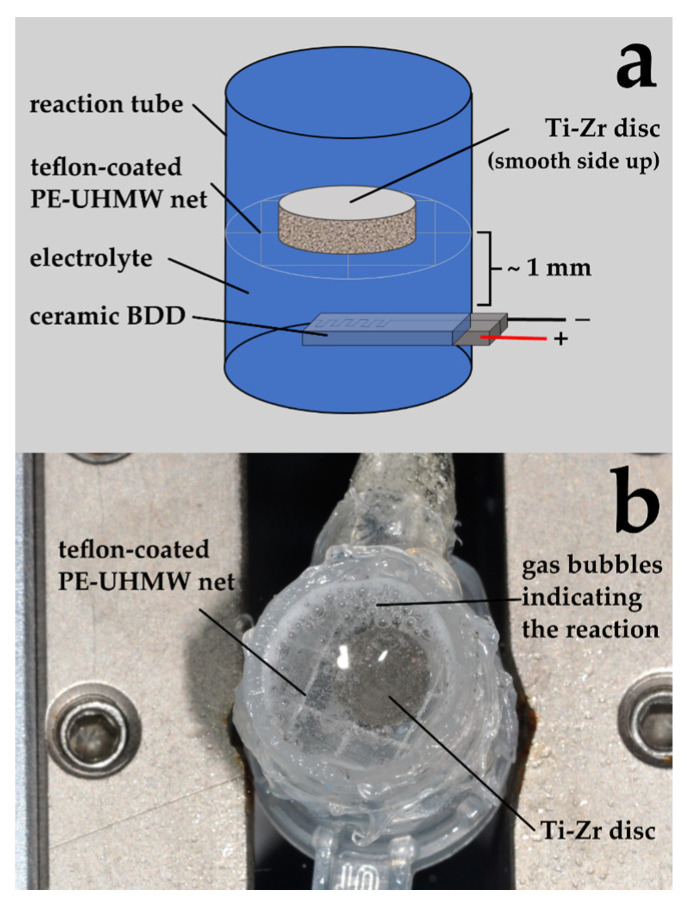
Experimental setup for electrochemical disinfection. (**a**) Schematic presentation, (**b**) situation during disinfection process with Ti-Zr disc resting on PE-UHMW net. Please note the gas bubbles indicating the electrochemical reaction.

From each volunteer, one disc was allocated to the treatment groups shown in Table 1. The treatment procedure is shown in Figure 3 as an example for chemo-mechanical debridement using curettes and chlorhexidine (Chlorhexamed FORTE ethanol-free 0.2%, GlaxoSmithKline Consumer Healthcare GmbH & Co. KG). The setup for the electrochemical debridement is shown in Figure 4. After treatment, discs were pressed on Columbia blood agar plates (Figure 3) and bacterial growth was analyzed (see below).

The apparatus for the electrochemical disinfection (Figure 4) was fabricated using teflon-coated polyurethane ultra-high molecular weight net (PE-UHMW—Deltex Mexxx Crystal; Trading House Müller e.K., Teterow, Germany), which was mounted inside a cut two milliliter reaction tube approximately 1 mm above the electrode. Both electrode and net were fixed with glue (Sofortkleber, Bindulin-Werk H.L. Schönleber GmbH, Fürth, Germany) and sealed with silicone (Aquarium-Dicht, Bindulin-Werk H.L. Schönleber GmbH, Fürth, Germany). Then, the electrode was connected to a DC power source (Voltcraft LPS 1305; Conrad Electronic AG, Wollerau, Switzerland).

For electrochemical disinfection, the roughened surfaces of the Ti-Zr discs faced the net inside the tube, and 1 mL of 0.9% NaCl solution was added (Figure 4). A potential of 6 V at an average current of 50 mA (Sample 1–3) or a potential of 9 V at an average current of 115 mA (Sample 4–6) was applied for 2.5 min and 5 min, respectively. The electrolyte was renewed for each sample, and the apparatus was rinsed with distilled water between experiments.

After their respective treatment, all samples were repeatedly pressed on Columbia blood agar (Oxoid, Thermo Fisher Scientific GmbH, Wesel, Germany) both with the rough and smooth surfaces (Figure 3d), and the blood agar plates were incubated for 24 h at 37 °C. After incubation, the plates were analyzed by an independent operator, not involved into the study, in order to avoid bias [37].

As an independent approach, implants were treated with the described electrode. A total of nine Straumann implants (BL 4.1 × 8 mm SLActive RC; REF: 021.4308, Straumann GmbH, Freiburg, Germany) were placed in reaction tubes filled with 4 mL Brain Heart Infusion (BHI, Oxoid, Wesel, Germany) and inoculated with three different multispecies mixtures (3 implants per mixture). The multispecies biofilms were allowed to grow for 24 h at 37 °C and 175 rpm. The suspension was removed, and the implants were rinsed with 5 mL of PBS. One implant per multispecies mixture was treated for 0 min (positive), 2.5 min, and 5 min submerged in PBS inside the disinfection apparatus described before. A potential of 9 V at an average current of 105 mA was applied to the electrode. Then, the implants were rolled on Columbia blood agar as described previously [34].

### 2.3. Biofilm Measurement

**Figure 5:**
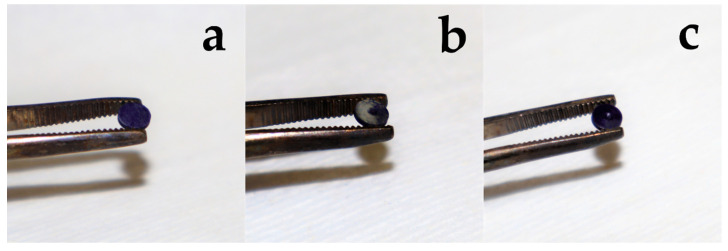
Crystal violet stained biofilm at the disc’s surface. (**a**) Roughened surface completely covered but also (**b**) mild and (**c**) strong biofilm formation at the smoother surface was observed.

Biofilm formation is characterized by the formation of a polymer matrix, typically consisting of sugars, sugar derivatives, and amino acid polymers into which the microorganisms are encapsulated. Using a crystal violet-based staining method, biofilm formation was determined. For this purpose, Ti-Zr discs were placed in 4 mL of BHI following the contact test on Columbia blood agar (see Section 2.2.2: Treatment of disc) and were incubated at 175 rpm for 24 h at 37 °C. The suspension was discarded, and the discs were washed with 4 mL of PBS and stained with 0.5 mL of Neisser II solution (Carl Roth GmbH + Co. KG, Karlsruhe, Germany) for 15 min. The stained discs were washed with deionized water until the excess dye was fully removed and dried afterwards. The stained biofilm (Figure 5) was eluted by 2 mL of 30% acetic acid, and the quantification was obtained via UV/Vis measurement at 570 nm using acetic acid as blank. Measurement and procedure of biofilm staining were derived from a procedure described previously [38].

### 2.4. Identification of Biofilm Microorganisms

Organisms colonizing the disc surfaces were isolated by streaking the six multispecies mixtures obtained from the positive control as well as the surviving organisms after DDE treatment on Columbia blood agar. Alternatively, colonies were resuspended in 4 mL of BHI followed by plating 100 µL of a dilution series on BHI agar plates. Single colonies were randomly picked and streaked-out twice to obtain pure cultures. A thin layer of bacteria from fresh colonies was applied to a stainless-steel target using a toothpick. HCCA matrix (1 µL, 10 mg/L α-cyano-4-hydroxycinnamic acid in 50% acetonitrile, 47.5% water, and 2.5% trifluoroacetic acid) was added to the bacterial film and dried at room temperature. The identification was performed by MALDI-ToF-MS using a Microflex LT™ and the Biotyper™ 3.1 Software (Bruker Daltonik GmbH, Bremen, Germany) using the “smart” method based on 40–200 single spectra.

## 3. Results

### 3.1. Multipecies Biofilm Formation

No adverse event occurred during the clinical phase of the experiment, and all Ti-Zr discs could be harvested and used for the disinfection experiments. As described in a recent study by Conserva et al. [37], biofilm formation occurred regardless of the surface roughness (Figure 5), while older studies [39] described a threshold value of surface roughness R(a) of 0.2 µm.

#### Composition of Natural Biofilms

**Figure 6:**
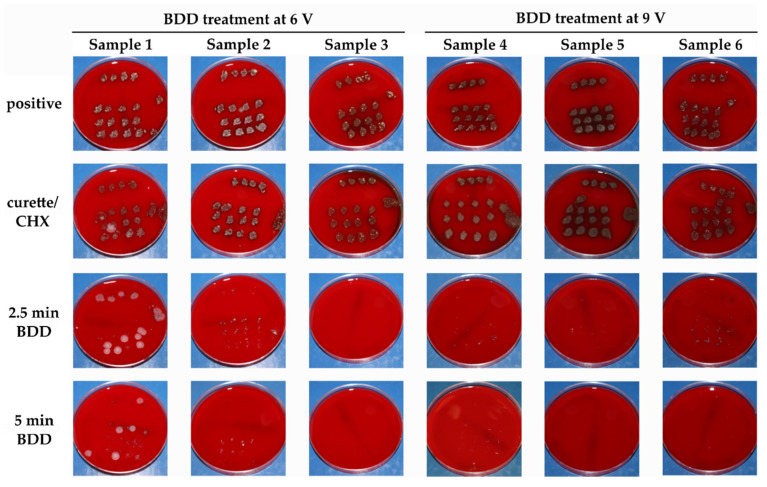
Images of blood agar plates into which the Ti-Zr discs had been repeatedly pressed. The plates have been incubated for 24 h at 37 °C.

Biofilm formation was achieved in all volunteers as shown by bacterial growth on Columbia blood agar plates (Figure 6). From these multispecies biofilms, single colony streak-outs were generated, and selected purified colonies were subjected to MALDI-ToF mass spectrometry for the identification of microorganisms. The results obtained revealed the presence of characteristic bacteria of the oral microbiome and showed individual variations (Table 2).

*Neisseria*, *Staphylococcus*, and *Streptococcus* species were most abundant followed by other Gram-negative and Gram-positive bacteria such as *Bacillus subtilis*, *Gemella haemolysans*, *Lactobacillus paracasei*, *Micrococcus luteus*, *Rothia dentocariosa*, and *Rothia mucilaginosa*. Yeasts such as Candida species were observed on the plates in only one case (based on their characteristic colony morphology) or in the random samples used for mass spectrometry. Interestingly, a high portion of the isolates were hemolytically active. α-Hemolysis was observed in case of *Streptococcus cristatus*, *Streptococcus gordonii*, *Streptococcus mitis*, *Streptococcus oralis*, *Streptococccus parasanguinis*, *Streptococcus peroris*, *Streptococcus salivarius*, *Streptococcus sanguinis*, and *Streptococcus vestibularis*. β-hemolysis was detected for *G. haemolysans* and *Staphylococcus haemolyticus*.

### 3.2. Elimination of Microorganisms 

#### 3.2.1. Inactivation of Bacterial Colonization by Mechanical Debridement and Electrochemical Disinfection

In none of the cases was mechanical debridement using plastic curettes and chlorhexidine (CHX) irrigation successful in removing the majority of microbes from the samples. With all Ti-Zr impressions on the blood agar plates showing bacterial growth, obviously no difference existed compared to no treatment. In Samples 1 and 2, bacterial growth was seen with all treatments rendered. Already after 2.5 min of DDE application, complete disinfection could be achieved in two cases (Samples 3, 6 V; Sample 5, 9 V), while three samples showed remaining minor bacterial growth. This situation further improved after 5 min of DDE application in complete disinfection in three cases (Samples 4–6, 9 V) and minor bacterial growth in two cases. Double diamond electrodes led to reduced growth as compared to the use of curettes and longer treatment times, and a higher potential had a positive effect as well. 

#### 3.2.2. Surviving Microorganisms

Microorganisms surviving DDE treatment—at least with low abundance—were also subjected to MALDI-ToF mass spectrometry. Treatment-resistant colony-forming units were members of *B. subtilis*, *Neisseria* sp., *R. mucilaginosa*, *S. haemolyticus*, *S. mitis*, *S. oralis*, and *S. salivarius* (Table 3).

#### 3.2.3. Removal of Biofilm 

**Figure 7:**
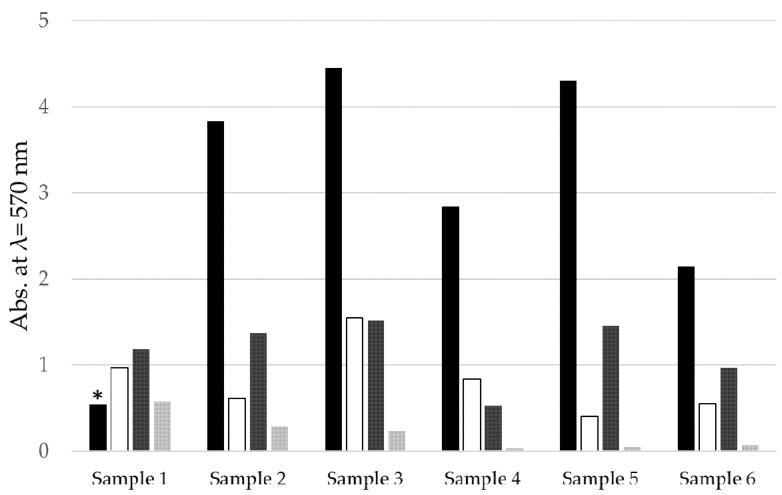
Quantitative analysis of biofilm formation at the Ti-Zr disc surface at different stages. Black: Control/no treatment/positive, white: curette/chlorhexidine, dark gray: 2.5 min DDE treatment, gray: 5 min DDE treatment, *: biofilm detached during washing step.

Biofilm matrices contribute significantly to the high resistance of biofilm-producing microorganisms against different kinds of abiotic and biotic stresses. This may explain the high resistance of the biofilm-forming bacteria to chlorhexidine. In the experiments described above, the inactivation of microorgansims was monitored. In addition, we quantified the biofilm itself, without distinguishing between bacteria and matrix (Figure 7). 

Biofilm mass was highest without treatment (with the exception of Sample 1, where biofilm was lost during staining). Combined curette and chlorhexidine treatment reduced up to seven-eighths of the biofilm (see Sample 5). An almost complete removal of biofilm was obtained by DDE treatment for five minutes. The data suggest not only an effective inactivation of microbes by DDE treatment, but also an elimination of matrix compounds at least in the case of fresh biofilms.

#### 3.2.4. BDD Treatment of Implants

**Figure 8:**
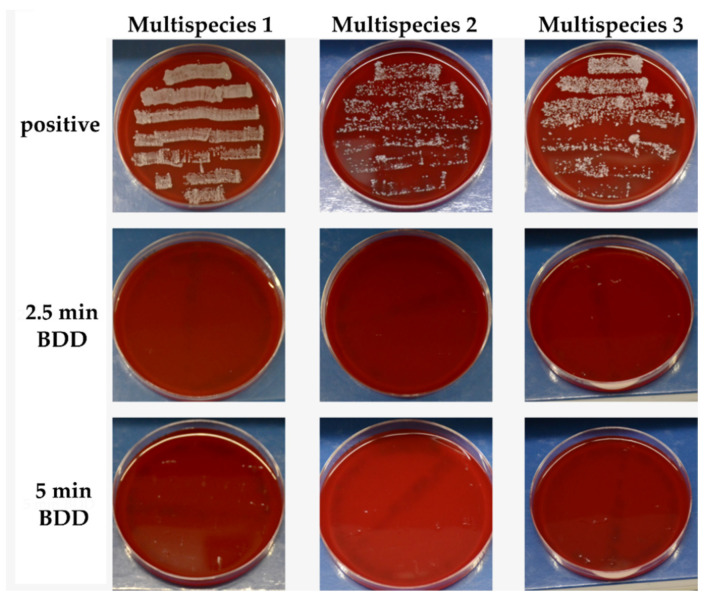
Standardized photographs of blood agar plates onto which the implants had been rolled. The plates have been incubated for 24 h at 37 °C. No bacterial growth was observed after DDE treatment at 9 V/Ø 105 mA.

All the multispecies mixtures used were able to form a biofilm at the implant surface after 24 h of incubation as shown by the roll-outs obtained from the positive samples. Already after 2.5 min of electrochemical disinfection in PBS, none of the multispecies biofilms remained active (Figure 8).

## 4. Discussion

This investigation compared chemo-mechanical debridement and electrochemical disinfection for eliminating wild-type biofilm from roughened Ti-Zr discs. The traditional approach of manually cleaning a metal surface in addition to rinsing was not effective in eliminating biofilm, which seems to be in line with previous reports [12,13]. While it may be argued that alternative treatment strategies such as air powder abrasion [15] may have been more appropriate as control, it has to be kept in mind that simple, flat surfaces were considered here, which could be well accessed. This is in contrast to clinical reality, where access for removing biofilm due to surface roughness and the macrodesign of implants [25,27] as well as defect morphology [26] is critical. The double diamond electrodes were kept at a distance of 1 mm to the disc surfaces and despite that, they were at least as effective in biofilm removal as the use of curettes. From this point of view, it might be more appropriate as a treatment option to inactivate bacteria with the body’s immune system removing the remaining bacterial components. At the current stage, the complete and permanent elimination of bacteria from implant surfaces seems to be impossible, and it is unknown whether or not the complete elimination of microbiota is necessary. The non-surgical application of the BDD electrode during maintenance visits would assist in reducing the bacterial load. As such, regular supportive therapy, which has been shown to be important for long-term implant success [4], would become possible with the double diamond electrode, which can be inserted in the peri-implant sulcus.

Currently, the described double diamond electrode is at an early stage of development requiring several questions to be answered prior to clinical application. These include the application of a fluid flow model as the amount of electrolyte is kept at a constant level in the current setup. In a clinical situation, the electrolyte would be rinsed over the electrode and implant with only minor accumulation being possible. The disinfectant effect may change if the electrolyte is constantly replaced, leading to a lower concentration of oxidants based on the total volume.

The radicals produced during electrochemical disinfection might also harm host tissue as they non-specifically interact with proteins, lipids, and nucleic acids [40,41]. Consequently, the next steps will involve the examination of specific treatment parameters necessary for optimal disinfection while not causing harm to host tissue. To meet this requirement within a medically appropriate time interval, an adjustable current output of the electrode while working at similar conditions (voltage, type of electrolyte) is necessary. Although 3 × 5 mm^2^ of the electrodes surface area may be electrochemically active, it can be assumed that most of the disinfectants are produced at the laser-structured area between the anode and cathode, which is 22 mm in length in this case. Altering the length by choosing a more compressed structure would result in a significant increase of the electric current, ultimately leading to a higher production of reactive oxygen species and consequently to a shorter treatment time. Considering this, the size of the presented electrode surface is not a limiting factor for sufficient disinfectant supply. In contrast, the BDD layer thickness, its degree of doping, and the distance between the anode and cathode may have a much higher impact on the disinfection performance of the electrode. Accordingly, the novel electrode type can be further reduced in size and adapted to application-specific requirements because of the geometry-independent diamond coating. Attention must be paid to the fact that ceramics usually fail by brittle fracture, which makes them more difficult to handle. The electrical contact and sealing have proven to be sufficient for the tests in this study, but they need to be revised in the future if the desired size of the electrode decreases.

Due to the design of the current experiments, several limitations have to be kept in mind when interpreting the results presented. Flat metal discs were used for the intraoral formation of biofilm, while in a clinical situation, more complex geometries of dental implants and prosthetic components are present. This was considered during the disinfection of dental implants where the more complex surface structure did not prevent complete electrochemical disinfection. However, the specimens were well accessible for both treatment options, while in a clinical setting, bony defects, prosthetic restorations, and patient-specific factors limit access. The current experiment was designed as a pilot study evaluating a novel electrode array. A limited sample size did not allow for meaningful comparative statistical analysis with sufficient power.

Organisms isolated from the sample surface are typically found in the oral cavity. The members of *Neisseria*, *Rothia*, *Staphylococcus*, and *Streptococcus* species found in this study are present in isolates from dental and peri-implant disease but are also often associated with a healthy oral status [42,43,44,45,46,47,48,49]. Almost 50% of the identified microorganisms in this study are *Streptococcus* species, which is not surprising as they play a major role beside *Actinomyces* in the initiation of surface colonization in the oral cavity [50]. Desch et al. also identified streptococci as a dominant part of the colonization at Ti and Zr surfaces in an in vivo study, especially during the first hours of biofilm formation, followed by *Neisseria*, *Rothia*, or *Gemella* [49]. These first colonizers are crucial for a later succession of the biofilm toward disease-causing conditions [51]. Consequently, the inactivation of these early-stage colonizers as well as a removal of the formed early biofilm can prevent more serious infections. *M. luteus* as well as *B. subtilis* are typical environmental bacteria and may have been contaminations resulting from the colonization procedure of the discs surface as the splints were removed during eating. Nevertheless, it has to be taken into account that the microflora present in healthy subjects cannot reflect the situation of dental implants affected by peri-implantitis. As such, the efficacy of double diamond electrodes can ultimately only be judged in clinical cases following final development and certification.

## 5. Conclusions

BDD electrode application was more effective in eliminating wild-type bacterial biofilm from Ti-Zr discs as compared to conventional treatment using mechanical debridement and CHX irrigation. While longer treatment time had a positive effect on disinfection, the complete elimination of bacteria could not be achieved in all cases, but at least after five minutes of treatment time using a potential of nine volts and an average current of 115 mA. The electrode used had overall dimensions that are clinically applicable, and the microstructuring process creating a double-electrode on one single carrier was successful. The charge quantity used for disinfection has to be optimized with respect to bacterial elimination and tissue preservation.

## 6. Patents

Stefan Rosiwal and Andreas Burkovski have filed a patent for the disinfection method described in this report.

## Figures and Tables

**Table 1:** Treatment groups.

Group	Abbreviation	Treatment
Control	Positive	No treatment
Chemo-mechanical debridement (Figure 3)	Curette	Mechanical debridement using plastic curettes and irrigation with chlorhexidine for 30 sec
Electrochemical disinfection (Figure 4) [32,34]	2.5 min BDD	Immersion in physiological NaCl solution and electrochemical disinfection for 2.5 min at 6 V (Sample 1–3) or 9 V (Sample 4–6)
	5 min BDD	Immersion in physiological NaCl solution and electrochemical disinfection for 5 min at 6 V (Sample 1–3) or 9 V (Sample 4–6)

**Table 2:** Microorganisms isolated from the roughened surface of untreated and double diamond electrode (DDE)-treated Ti-Zr discs. Identification was performed via MALDI-ToF analysis (bold numbers indicate a Score > 2.3 = highly probable species identification, Score > 2.0 = secure genus, probable species identification).

Organisms	Score
**Sample 1**	
*Bacillus subtilis*	2.251
*Micrococcus luteus*	2.086
*Neisseria flavescens*	2.187
*Neisseria* sp.	2.171
*Rothia mucilaginosa*	2.267
*Staphylococcus haemolyticus*	**2.326**
*Streptococcus cristatus*	2.142
*Streptococcus oralis*	2.11
*Streptococcus mitis*	**2.37**
*Streptococcus sanguinis*	2.151
**Sample 2**	
*Micrococcus luteus*	2.173
*Neisseria mucosa*	2.111
*Neisseria* sp.	2.231
*Staphylococcus epidermidis*	2.082
*Staphylococcus haemolyticus*	**2.382**
*Staphylococcus succinus*	2.047
*Streptococcus oralis*	2.062
*Streptococcus parasanguinis*	2.082
*Streptococcus salivarius* ssp *salivarius*	2.171
*Streptococcus vestibularis*	2.224
**Sample 3**	
*Neisseria* sp.	2.145
*Rothia dentocariosa*	2.277
*Streptococcus mitis*	2.257
*Streptococcus oralis*	2.118
**Sample 4**	
*Candida albicans*	2.21
*Lactobacillus paracasei*	**2.432**
*Mirococcus luteus*	2.212
*Neisseria subflava*	2.196
*Rothia mucilaginosa*	2.281
*Streptococcus mitis*	2.258
*Streptococcus oralis*	**2.328**
*Streptococcus salivarius*	2.281
*Streptococcus salivarius* ssp. *salivarius*	**2.318**
*Streptococcus vestibularis*	**2.365**
**Sample 5**	
*Gemella haemolysans*	**2.323**
*Lactobacillus paracasei*	**2.376**
*Micrococcus luteus*	2.284
*Neisseria mucosa*	2.247
*Streptococcus gordonii*	2.188
*Streptococcus mitis*	**2.308**
*Streptococcus oralis*	**2.321**
**Sample 6**	
*Micrococcus luteus*	2.287
*Neisseria flavescens*	2.247
*Rothia mucilaginosa*	2.27
*Staphylococcus epidermidis*	2.102
*Staphylococcus haemolyticus*	1.95
*Streptococcus oralis*	2.292
*Streptococcus peroris*	2.091
*Streptococcus salivarius*	**2.333**

**Table 3:** Bacteria identified using MALDI-ToF and listed according their maximum resistance against DDE treatment.

**Sensitive at 6 V**	**Survival of 2.5 min of DDE Treatment**	**Survival of 5 min of DDE Treatment**
*Neisseria flavescens* *Neisseria mucosa* *Rothia dentocariosa* *Staphylococcus epidermidis* *Staphylococcus succinus* *Streptococcus christatus* *Streptococcus oralis* *Streptococcus parasanguinis* *Streptococcus salivarius* ssp. *salivarius* *Streptococcus sanguinis* *Streptococcus vestibularis*	Not tested	*Bacillus subtilis* *Neisseria* sp. *Rothia mucilaginosa* *Staphylococcus haemolyticus* *Streptococcus mitis*
**Sensitive at 9 V**	**Survival of 2.5 min of DDE Treatment**	**Survival of 5 min of DDE Treatment**
*Candida albicans* *Gemella haemolysans* *Lactobacillus paracasei* *Neisseria flavescens* *Neisseria mucosa* *Neisseria subflava* *Staphylococcus epidermidis* *Staphylococcus haemolyticus* *Streptococcus gordonii* *Streptococcus mitis* *Streptococcus peroris* *Streptococcus salivarius* ssp. *salivarius* *Streptococcus vestibularis*	*Streptococcus oralis* *Streptococcus salivarius* *Rothia mucilaginosa*	No surviving microorganisms.
